# Striking Differences between Knockout and Wild-Type Mice in Global Gene Expression Variability

**DOI:** 10.1371/journal.pone.0097734

**Published:** 2014-05-15

**Authors:** Satish A. Eraly

**Affiliations:** Department of Medicine, University of California San Diego, School of Medicine, La Jolla, California, United States of America; Radboud University, Netherlands

## Abstract

Microarray analyses of gene knockouts have traditionally focused on the identification of genes whose *mean* expression is different in knockout and wild-type mice. However, recent work suggests that changes in the *variability* of gene expression can have important phenotypic consequences as well. Here, in an unbiased sample of publicly available microarray data on gene expression in various knockouts, highly significant differences from wild-type (either increases or decreases) are noted in the gene expression coefficients of variation (CVs) of virtually every knockout considered. Examination of the distribution of gene-by-gene CV differences indicates that these findings are not attributable to a few outlier genes, but rather to broadly increased or decreased CV in the various knockouts over all the (tens of thousands of) transcripts assayed. These global differences in variability may reflect either authentic biological effects of the knockouts or merely experimental inconsistencies. However, regardless of the underlying explanation, the variability differences are of importance as they will influence both the statistical detection of gene expression changes and, potentially, the knockout phenotype itself.

## Introduction

As with other biological perturbations of interest, the effects of gene knockouts have conventionally been considered in terms of the changes elicited in the *average* quantity of various measurable attributes. Thus, microarray (and other transcriptome-level) analyses of gene expression in knockout mice have focused on the identification of genes whose average RNA expression is significantly altered from that in wild-type mice. From this perspective, *variability* in gene expression is principally of interest as a component of the statistical tests used to assess the significance of changes in average expression.

However, recent research supports the notion that such variability may be, in and of itself, a significant determinant of phenotype. For instance, changes in gene expression variability have been associated with several human diseases, including schizophrenia, Parkinson's disease, muscular dystrophy, dilated cardiomyopathy, and lung and colorectal adenocarcinoma [1], [2]. Indeed, hyper-variability of gene expression may be a general property of malignancies [3], [4], providing the basis for novel variability-based cancer diagnostics [3]. Moreover stochastic variation in gene expression may help drive developmental processes, by, e.g., generating heterogeneity in an initially homogenous cell population, thereby permitting differentiation [4], [5], [6]. Notably, the intermediary steps linking increased gene expression variability to an intestinal developmental defect in *C. elegans* have been delineated in molecular detail [7].

Here marked differences between gene knockout and wild-type mice in global gene expression variability are noted in an unbiased sample of publicly available microarray data. These differences may derive from actions of the genes knocked out or simply from dissimilar experimental handling of the knockout compared with wild-type mice. In either case, though, they have clear implications for both the statistical analyses of knockout microarray data and, importantly, the knockout phenotype itself.

## Results

**Figure 1:**
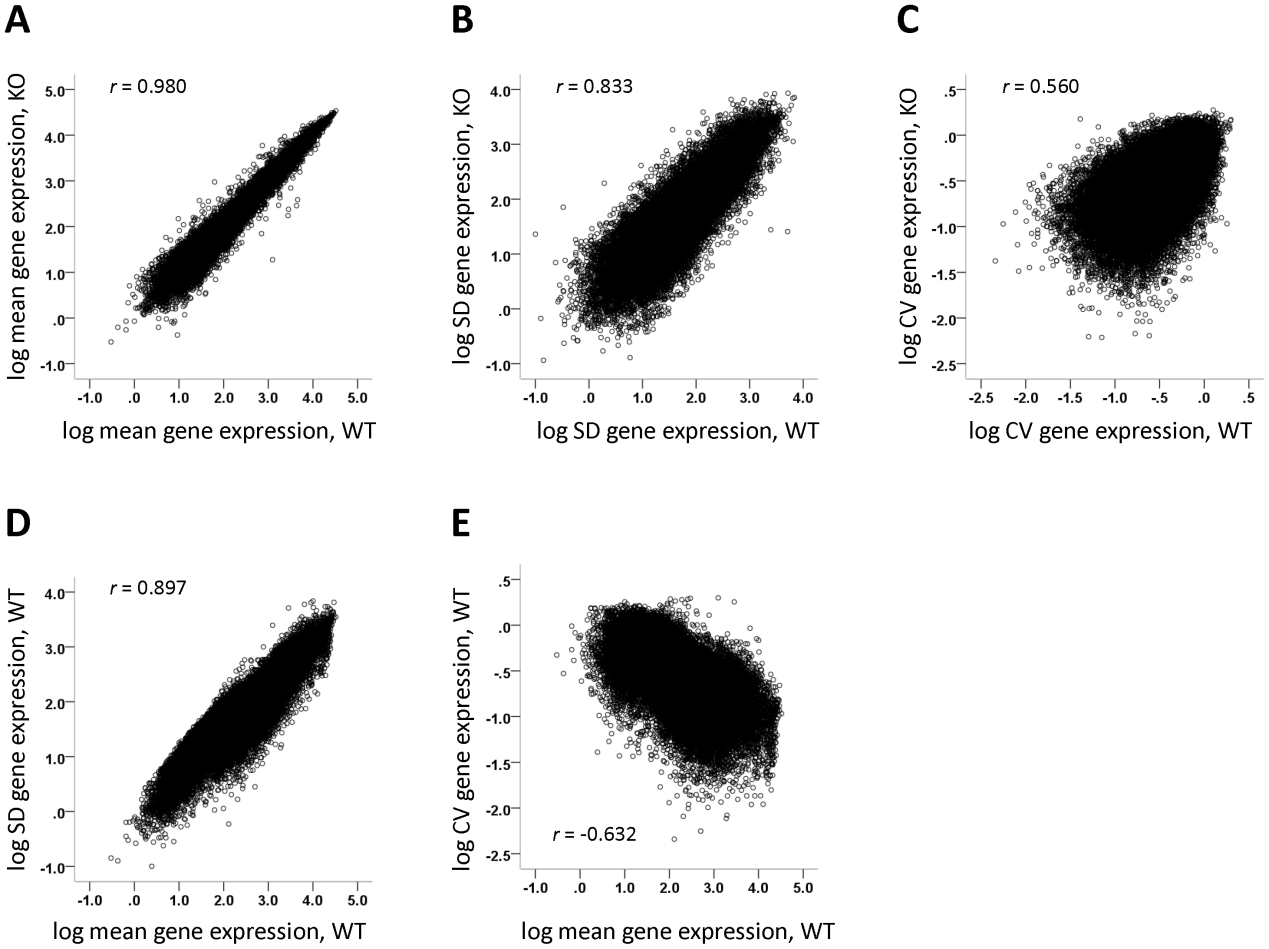
Relationships between sample statistics in a representative microarray dataset of gene expression in wild-type and knockout mice (#19 in Table 1). A, Log mean gene expression (log mean value for each of the ∼45,000 probe sets on the microarray) in knockout vs. log mean in wild-type. B, Log standard deviation (SD) of gene expression in knockout vs. log SD in wild-type. C, Log CV of gene expression in knockout vs. log CV in wild-type. D, Log SD of expression vs. log mean in wild-type. E, Log CV of expression vs. log mean in wild-type. SD, standard deviation; KO, knockout; WT, wild-type; *r*, Pearson's correlation coefficient.

Microarray datasets selected for analysis (see Methods for the inclusion criteria) are listed in Table 1. As anticipated (given that significantly differentially expressed genes constitute a negligible fraction in typical microarray experiments), levels of gene expression in knockouts were tightly correlated with those in wild-types in these datasets (Fig. 1A). Standard deviation in knockouts was also highly correlated with that in wild-types (Fig. 1B). The coefficient of variation (CV) is the ratio of the standard deviation to the mean. The statistical errors in the mean and standard deviation jointly contribute to the error in their ratio, so that the correlation of knockout to wild-type CV was expectedly lower (Fig. 1C). Examining the relationship between gene expression level and variability, standard deviation was noted to essentially increase linearly with mean expression as indicated by the tight positive correlation between these variables (Fig. 1D). However, when evaluating *relative* variability, there was a *negative* correlation between mean gene expression level and CV (Fig. 1E). This latter relationship is presumably attributable to “noise” that does not vary with gene expression, and which therefore contributes greater variability relative to the mean at low than at high expression levels.

**Table 1:** Gene Expression Omnibus (GEO) datasets analyzed in this paper, ordered by increasing mean △log CV (see Fig. 2).

Dataset #	GEO name	Gene knocked out	RNA source	Repli-cates*	Reference	Use of litter-mates**
1	GSE22989	TGFBR2 (transforming growth factor β receptor, type II)	embryonic palate	5	[15]	
2	GSE7676	Pcdh12 (protocadherin 12)	placenta	5	[16]	y
3	GSE31940	Pex5	gonadal adipose	4	unpublished	
4	GSE41558	SRC-2 (steroid receptor coactivator-2)	heart	4	[17]	y
5	GSE12609	Arx (aristaless-related homeobox)	embryonic brain	4	[18]	
6	GSE24683	Dicer	adipose	4	[19]	
7	GSE47205	Mll (mixed lineage leukemia histone methyltransferase)	hematopoietic stem cells	5	[20]	
8	GSE16381	Txnrd1 (thioredoxin reductase 1)	liver	4	[21]	
9	GSE18326	FoxO3	forebrain neural stem cells	4	[22]	
10	GSE27309	sirtuin 3	brown adipose	5	[23]	y
11	GSE17985	Dicer	oocytes	4	[24]	
12	GSE8269	COX-1 (cycloxygenase 1)	gestational uterus	4	[25]	
13	GSE13807	Dicer	embryonic mouse limbs	5	unpublished	y
14	GSE31958	Cryptochrome 1 & 2	embryonic fibroblasts	4	[26]	
15	GSE8555	Phgdh (D-3-phosphoglycerate dehydrogenase)	embryonic head	4	[27]	y
16	GSE10895	MFP-2 (D-specific multifunctional protein 2)	liver	4	[28]	y
17	GSE15349	myostatin	muscle	5	[29]	
18	GSE9123	PlagL2 (pleomorphic adenoma gene-like 2)	embryonic small intestine	4	[30]	
19	GSE3843	Glycerol kinase	liver	4	[31]	y
20	GSE11899	Dicer	liver	5	[32]	
21	GSE9012	Trim24 (TIF1alpha)	liver tumor	5	[33]	y
22	GSE7424	GalT (α1,3 galactosyltransferase)	transplanted heart	4	[34]	
23	GSE27630	Otx2	choroid plexus	4	[35]	
24	GSE38988	COX-2 (cycloxygenase 2)	pancreas	4	[36]	
25	GSE7020	Nix	spleen	4	[37]	

*Number of biological replicates

**“y” denotes datasets for which either the GEO annotation or the relevant publication included a specific statement that the knockout and wild-type mice compared were littermates (in the case of the other datasets, the use or not of littermates was not specified).

In order to ascertain any differences in overall gene expression variability, the ∼45,000 CVs of gene expression in knockouts were compared to those in wild-types for each of the 25 datasets. (CVs rather than standard deviations were used in order to make variability comparable across genes with different levels of expression.) Every CV determined in the knockouts “matched” one determined for the same probe set in the wild-types. Accordingly, the overall variability difference between knockouts and wild-types in a dataset was quantified by determining the fold-change in CV (knockout CV/wild-type CV) for each probe set and then taking the geometric mean of these fold- changes across all ∼45,000 probe sets. (Fold-change was used in preference to the arithmetic difference in CV so as to avoid overweighting of higher CV-probe sets. As described further below, comparable results to those reported here were obtained when arithmetic differences rather than fold-changes were used.). These data were log-transformed in order to render them amenable to standard statistical approaches, so that the fold-changes in CV were represented by the differences between knockouts and wild-types in log CV, hereafter termed △log CV, and the geometric mean of the fold-changes by the arithmetic mean of △log CV. Finally, since individual tissues are estimated to ordinarily express only about half or fewer of the protein-coding sequences in their genomes, ([9], [10], [11]; and see especially [12], [13] for estimates derived from hybridization to Affymetrix microarrays), probe sets in each dataset that had expression values below the median (see *Methods*) were filtered out as likely representing genes not actually expressed and thus liable to contribute only noise to the analyses. (As described below, generally comparable results were obtained when all probe sets were included rather than just those with expression greater than the median.)

**Figure 2:**
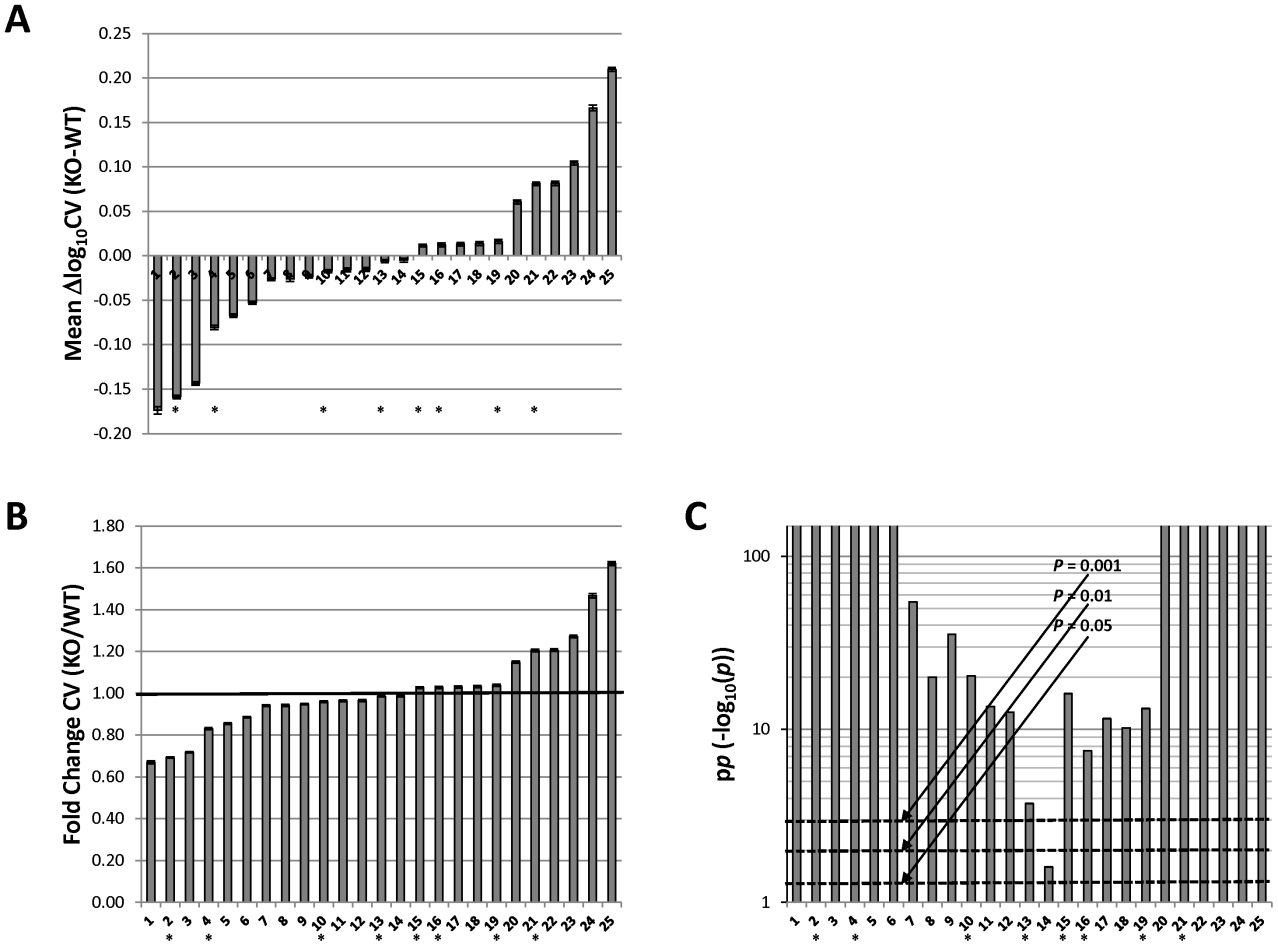
Differences between knockout and wild-type mice in global gene expression variability. A, Mean (across the ∼45,000 probe sets) of the differences (△) between knockout and wild-type in log_10_ CV in each of the 25 datasets analyzed. B, Mean fold change (knockout/wild-type) in CV in the 25 datasets (obtained by exponentiating the corresponding mean △log_10_ CV values). C, Statistical significance of mean △log_10_ CV in the 25 datasets, as indicated by the negative log_10_ of the corresponding *p* values (termed p*p* here). (Higher p*p* values correspond to lower *p*; e.g., a p*p* value of 10 indicates *p* = 10^−10^ and a p*p* value of 100 indicates *p* = 10^−100^.) Dashed lines denote the usual thresholds of statistical significance: *p* = 0.05 (corresponding to p*p* = 1.30), *p* = 0.01 (p*p* = 2), & *p* = 0.001 (p*p* = 3). Note that the p*p* values are themselves presented on a log scale so as to enable comparison across their entire range. Numbering of datasets is as in Table 1: 1, TGFBR2; 2, Pcdh12; 3, Pex5; 4, SRC-2; 5, Arx; 6, Dicer; 7, Mll; 8, Txnrd1; 9, FoxO3; 10, sirtuin 3; 11, Dicer; 12, COX-1; 13, Dicer; 14, Cryptochrome 1 & 2; 15, Phgdh; 16, MFP-2; 17, myostatin; 18, PlagL2; 19, Glycerol kinase; 20, Dicer; 21, Trim24; 22, GalT; 23, Otx2; 24, COX-2; 25, Nix. Error bars in panels A & B denote SEM. △log_10_ CV, difference between knockout and wild-type in log_10_ CV; KO, knockout; WT, wild-type; *, datasets affirming use of littermate controls (see Table 1). Please see the text for details.

These investigations revealed marked differences between the knockout and wild-type mice in global gene expression variability. While datasets were roughly evenly divided between those in which knockouts manifested decreased variability (14 of 25 datasets) and those in which their variability was increased (11 datasets) – with mean +/− SEM of △log CV ranging from −0.174 +/− 0.004 to 0.210 +/− 0.002 (corresponding to mean fold-changes in CV (knockout/wild-type) of 0.67 and 1.62, respectively) (Figs. 2A & 2B) – in virtually every dataset the change in variability was highly statistically significant (Fig. 2C): The *p* value for △log CV was <0.05 in all the datasets, <0.001 in 24 of the 25 datasets, <10^−7^ in 23, <10^−10^ in 22, and <10^−100^ in 12, i.e., almost half the datasets. Comparable results were obtained when differences in CV (△CV) were considered rather than △log CV; for instance, the p value for △CV was <10^−10^ in 22 of the 25 datasets and <10^−100^ in 10 datasets. Similarly, generally comparable results were obtained when all genes were included rather than just those with expression greater than the median: the p value for △log CV in such analyses was <10^−10^ in 19 of the 25 datasets and <10^−100^ in 13 datasets.

If these highly significant differences in variability were attributable to outliers (genes for which the CV in the knockout was greatly different from that in the wild-type), with the variability of the remaining genes not systematically altered, then it would be expected that the distributions of the ∼45,000 △log CV values in the various datasets would have their center (median) at approximately zero, with the mean “tugged” by the outliers in a negative or positive direction (for datasets towards the left and right end, respectively, of Fig. 2). On the other hand if the variability differences reflected broadly increased or decreased CV in the knockouts over most of the assayed genes, then the medians of the △log CV distributions would tend to concur with the means.

**Figure 3:**
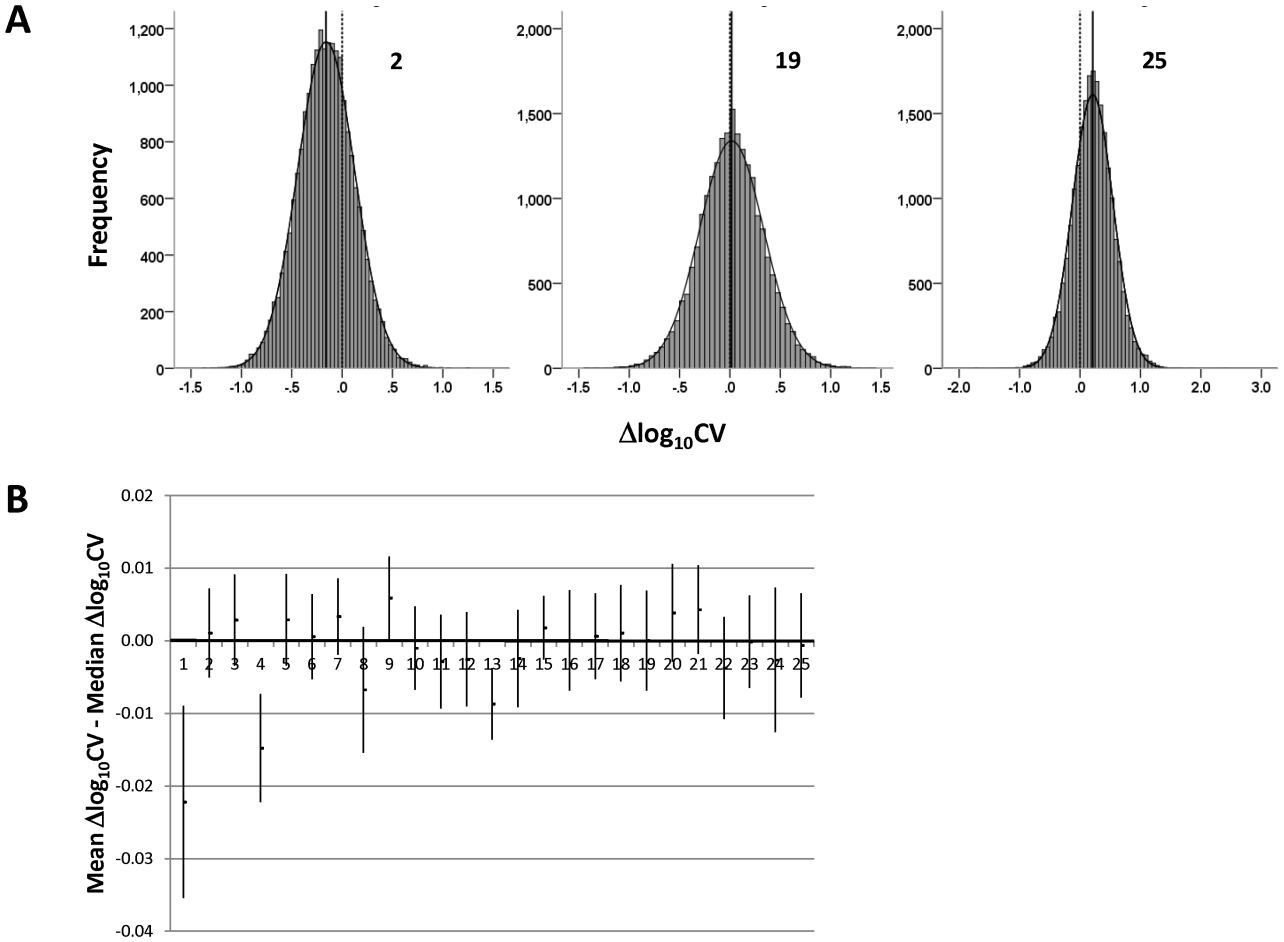
Characteristics of △log_10_ CV distributions. A, Distribution of △log_10_ CV in three representative datasets (#2, #19, & #25 in Table 1). Normal curves are superimposed on the histograms; the centers of these curves indicate the positions of the means of the distributions. The solid vertical lines pass through the medians of the distributions and the dashed vertical lines through zero. B, 95% confidence intervals for the differences between the means and medians in the 25 datasets. Numbering of datasets is as in Table 1: 1, TGFBR2; 2, Pcdh12; 3, Pex5; 4, SRC-2; 5, Arx; 6, Dicer; 7, Mll; 8, Txnrd1; 9, FoxO3; 10, sirtuin 3; 11, Dicer; 12, COX-1; 13, Dicer; 14, Cryptochrome 1 & 2; 15, Phgdh; 16, MFP-2; 17, myostatin; 18, PlagL2; 19, Glycerol kinase; 20, Dicer; 21, Trim24; 22, GalT; 23, Otx2; 24, COX-2; 25, Nix. Please see the text for details.

This latter scenario was what was in fact observed: △log CV distributions, which were largely Gaussian, had their medians mostly approximately coinciding with their means rather than with zero. The distributions of three representative datasets are illustrated in Fig. 3A with the positions of the median (solid vertical line), mean (midpoint of the superimposed normal curve), and zero (dashed vertical line) indicated. The correspondence between the medians and means in the 25 datasets was quantified by computing 95% confidence intervals for the differences between them (Fig. 3B); in 22 datasets this interval spanned zero, indicating no statistically significant difference between median and mean. Moreover, in the case of two of the three datasets that did manifest significant differences between median and mean, the median was nevertheless much closer to the mean than to zero, arguing against an overriding contribution of outliers in even these datasets: the medians and means were −0.151 and −0.174, respectively, in dataset #1 (numbering from Table 1), and −0.066 and −0.080 in #4 (values for median and mean in the third dataset, #13, which had a relatively low *p* value for △log CV (see Fig. 2C), were 0.003 and −0.006).

## Discussion

The findings presented here reveal striking differences between knockout and wild-type mice in the variability of their gene expression. These differences generally cannot be ascribed to the effects of a minority of genes with aberrant expression, but rather primarily reflect broadly increased or decreased variability in the knockouts compared with wild-types. How might the knockout of a gene influence overall gene expression variability? All else equal, decreasing the number of inputs to a system should reduce its stability (in the same way that decreasing the number of observations reduces the precision of the mean). In particular, genes mediating negative feedback would tend to buffer stochastic effects, so that their loss could increase variability. Conversely, the loss of genes participating in positive feedback loops or which are themselves subject to stochastic regulation might decrease variability [4], [5], [6]. The Gene Ontology (GO; www.geneontology.org; accessed August, 2013) annotations of the genes knocked out in the studies analyzed in this paper are displayed graphically in Fig. S1 to allow for visual comparison of functional categorizations across genes. Using the “Term Enrichment Tool” (www.geneontology.org; accessed August, 2013), there were no significant differences in annotation between the genes whose knockout was associated with decreased variability and those whose knockout was associated with increased variability (not shown). This suggests an absence of sweeping functional distinctions between these two sets of genes. Notably, knockout of Dicer was associated with both substantially decreased (dataset #6; knockout in adipose) and increased (dataset #20; knockout in liver) variability (Table 1 and Fig. 2). There was also no clear relationship between the expression levels of the knocked out genes (in the tissues in which the microarray analyses were conducted) and their associated variability changes (Fig. S2). Nevertheless, finer-grained distinctions may emerge when larger sets of genes are considered.

An alternate mechanism whereby knockout of a gene might affect gene expression variability is broad induction or repression of gene transcription. Because there is generally an inverse relationship between expression level and (measured) variability (see, e.g., Fig. 1E), transcriptional induction of a large number of genes might be expected to decrease global variability and transcriptional repression to increase it. Accordingly, the relationship across the datasets between the mean expression difference between knockouts and wild-types and mean variability difference was assessed. Mean variability differences were taken from the data in Figure 2 and mean expression differences for each dataset were calculated in an analogous manner as the mean across probe sets of the difference between log_10_ expression in knockout and log_10_ expression in wild-type, filtering out probe sets falling below a specified expression threshold (this is equivalent to calculating the geometric mean of the knockout/wild-type expression ratios of all expressed genes). No significant relationship was noted between the mean variability differences of datasets and their mean expression differences (Fig. S3; Pearson's correlation coefficient  = 0.063, *p* = 0.766, and Spearman's correlation coefficient  = 0.033, *p* = 0.87). This suggests that the observed variability differences cannot be explained by global changes in expression level.

It is also possible that the observed variability differences merely represent an artifact of inconsistencies in the experimental procedures used during generation of the microarray data. For example, wild-type and knockout mice might not be matched with respect to genetic background, which might occur most frequently when experimental animals were insufficiently back-crossed to render them congenic. Moreover, even if congenics were generated, if knockouts but not their wild-type controls were litter- and cage-mates, or vice versa, substantial differences between the genotypes in the variability (and also mean) of gene expression could arise. Of note, though, the findings remain materially the same if one were to consider only those eight of the 25 datasets for which there was an explicit statement (in either their GEO annotation or associated publication) that knockouts and wild-types were derived from the same (heterozygote-bred) litter (and thus were likely cage-mates) (the other 17 datasets neither asserted nor disclaimed the use of littermates): In all eight of these datasets (#2, #4, #10, #13, #15, #16, #19, and #21; numbering from Table 1) the *p* value for △log CV was <0.001; *p* was <10^−7^ in seven of the eight, <10^−10^ in six, and <10^−100^ in three (Table 1 and Fig. 2).

However, regardless of the reason for it, any altered variability in knockouts is of practical importance. The statistical significance of an observed difference in gene expression levels is generally ascertained by assessing the magnitude of the difference relative to the estimated variability of gene expression in the conditions being compared. Thus mis-estimation of gene expression variability will contribute to mis-identification of differentially expressed genes. Previously described statistical methods that can accommodate global variability differences [14] ought to prove useful in this regard.

Importantly, the effects of altered variability on the knockout phenotype itself may also need to be considered. While increased variability might prove deleterious in contributing to deviations from a homeostatic equilibrium, decreased variability might also prove deleterious in limiting the capacity to adapt to environmental fluctuations [4], [5], [6]. Moreover, since there is not necessarily (or even typically) a linear relationship between gene expression and phenotype, certain traits may emerge only at the extremes of the range of gene expression. Such traits would be observed more frequently under conditions of increased expression variability even if mean expression remained constant (this consideration would generally apply in the case of categorical as opposed to continuous outcomes). Clearly, an improved understanding of gene expression variability changes in knockout mice would be of use in disentangling their pathophysiology and, thus, their implications for human disease.

## Methods

### Selection of microarray datasets

Microarray datasets involving comparison of gene expression in knockout and wild-type mice were obtained in July 2013 from the Gene Expression Omnibus (GEO [8]; http://www.ncbi.nlm.nih.gov/geo), a public repository of high throughput functional genomic data. The Affymetrix Mouse Genome 430 2.0 microarray, was overwhelmingly the mouse sequence platform most frequently represented in GEO; datasets using this platform comprised over 34,000 independent experimental samples, more than the number comprised by the next five most frequently represented mouse platforms combined. Accordingly, for ease of comparisons across datasets, searches were restricted to this platform, termed GPL1261 in the GEO nomenclature. While the great majority of knockout-associated microarray experiments in GEO had three or fewer replicates, I sought to retrieve data with four or five biological replicates so as to achieve greater precision in the estimation of statistical parameters. Since GEO cannot be searched by the number of replicates, but only by the total number of samples in a dataset, I searched for datasets with eight or ten samples, corresponding, respectively, to four or five replicates in cases of simple comparisons of gene expression in wild-types and knockouts (but potentially to different numbers of replicates for other experimental designs). Finally, I used the keywords “knockout,” “null,” “deletion,” and “lacking,” to identify analyses of knockouts.

Thus, the final GEO query took the form: “GPL1261 AND (8 OR 10[Number of Samples]) AND (knockout OR null OR deletion OR lacking).” Of the 61 datasets located by this query, 34 were discarded for having fewer than four biological replicates or for not involving comparison of gene expression in knockout and wild-type tissue. Of the remaining 27, two additional datasets were discarded because ∼22% of gene expression values in one and ∼40% in the other were negative (none of the other datasets had any negative values), leaving 25 datasets, listed in Table 1, that were included in the analyses reported here.

### Calculations and statistics

The 430 2.0 microarray contains ∼45,000 “probe sets” (groups of array features derived from a common sequence) intended to represent up to 39,000 well-annotated transcripts (http://www.affymetrix.com; accessed July, 2013); following sample hybridization and image processing, each probe set generates a single data point denoting the relative expression level of the corresponding sequence. For each dataset analyzed, the mean, standard deviation, and coefficient of variation (CV; ratio of standard deviation to mean) of gene expression levels (probe set values) in wild-type and knockout tissue were computed for each of the ∼45,000 probe sets, followed by calculation, for each probe set, of the difference between wild-type and knockout in log_10_ CV, hereafter referred to as delta (△) log CV (see the *Results* for the rationale for this choice). The mean value (across the ∼45,000 probe sets) of △log CV was calculated and its significance computed using a one sample, two-tailed *t*-test (under the null hypothesis of no difference between wild-type and knockout in CV; i.e., △log CV = 0). (Note that the requirement for normality in applying the *t*-test is greatly mitigated here owing to the very large sample size (∼45,000); nevertheless, the distributions of △log CV in the various datasets were in fact approximately normal (see Fig. 3A).) These calculations were repeated after filtering out probe sets for which the mean expression in either wild-type or knockout was less than the median of gene expression across all probe sets (see the *Results* for rationale). Statistical analyses were performed with SPSS v21 (IBM, Armonk, NY).

## Supporting Information

Figure S1 **Gene Ontology (GO) annotations of the genes knocked out in the analyzed studies.** Annotations were obtained from the Stanford Source (http://source.stanford.edu) and GeneCards (http://www.genecards.org/), and manually curated. Genes are listed in the same order as the corresponding knockouts in Figure 2 (i.e., in the order of increasing associated variability difference).
(PDF)

Figure S2 **Relative gene expression values of the genes knocked out in the analyzed datasets.** Gene expression values of the knocked out genes in (the wild-type replicates of) the tissues in which the corresponding microarray analyses were conducted were obtained as follows: Probe sets on the Affymetrix 430 2.0 microarray corresponding to these genes were identified using NetAffx software (http://www.affymetrix.com/analysis) (with the exception of myostatin which is not represented on this microarray). Most genes were represented by multiple (two to four) probe sets. The relative expression value for each knocked out gene was then computed from data in the corresponding dataset as the average expression in wild-type replicates across all probe sets representing that gene, normalized to the median gene expression value in the dataset. Genes are listed in the same order as the corresponding knockouts in Figure 2 (i.e., in the order of increasing associated variability difference).
(PDF)

Figure S3 **Relationship across the twenty-five analyzed datasets between the mean expression difference between knockouts and wild-types and mean variability difference.** The mean expression difference for each dataset was calculated as the mean across probe sets of the difference between log10 mean expression in knockout and log10 mean expression in wild-type (termed here Δlog10mean gene expression (KO-WT)), filtering out probe sets falling below a specified expression threshold. Mean variability differences (termed here Δlog10CV (KO-WT)) were taken from the data in Figure 2 and were calculated in an analogous manner. No significant relationship was noted between the expression and variability differences (Pearson's correlation coefficient  = 0.063, *p* = 0.766; Spearman's correlation coefficient  = 0.033, *p* = 0.87). KO, knockout; WT, wild-type; CV, coefficient of variation. Note the different scales of the two axes. Please refer to the text for details.
(PDF)
