# Supplementary material for: Striking Differences between Knockout and Wild-Type Mice in Global Gene Expression Variability
Source: PLoS One. 2014 May 15;9(5):e97734. doi: 10.1371/journal.pone.0097734 (PMC4022672; doi:10.1371/journal.pone.0097734)
Supplement: Figure S1 — Gene Ontology (GO) annotations of the genes knocked out in the analyzed studies. Annotations were obtained from the Stanford Source (http://source.stanford.edu) and GeneCards (http://www.genecards.org/), and manually curated. Genes are listed in the same order as the corresponding knockouts in Figure 2 (i.e., in the order of increasing associated variability difference). (PDF) [file pone.0097734.s001.pdf]

	TGFBR2	Pcdh12	Pex5	SRC-2	Arx	Dicer	Mll	Txnrd1	FoxO3	sirtuin 3	Dicer	COX-1	Dicer	Cryptochrome 1 & 2	Phgdh	MFP-2	myostatin	PlagL2	Glycerol kinase	Dicer	Trim24	GalT	Otx2	COX-2	Nix
**MOLECULAR FUNCTION**																									
GENE EXPRESSION																									
histone modification							■			■															
transcription factor					■				■									■					■		
transcriptional coactivator				■																	■				
ribonuclease						■					■		■							■					
SIGNALING AND METABOLISM																									
cytokine																	■								
protein kinase	■																								
protein binding		■		■										■							■				■
protein transport			■											■											
oxidoreductase								■				■			■	■								■	
transferase (not protein kinase)																			■			■			
**BIOLOGICAL PROCESS**																									
GENE EXPRESSION																									
chromatin silencing										■															
regulation of transcription				■	■		■		■	■				■				■			■		■		
RNA processing						■					■		■							■					
SIGNALING AND METABOLISM																									
prostaglandin biosynthesis												■												■	
regulation of metabolism			■	■		■			■	■	■		■			■		■	■	■		■			
lipid metabolism			■							■		■				■								■	
amino acid biosynthesis															■										
oxidative stress								■		■															
ubiquitination																					■				
GROWTH AND DEVELOPMENT																									
stem cell maintenance						■			■		■		■							■					
regulation of cell proliferation	■																■				■				
cell adhesion		■																							
development & differentiation	■			■	■	■	■		■		■		■		■		■			■			■		
apoptosis						■			■	■	■		■					■		■					■
**CELLULAR COMPONENT**																									
extracellular																	■								
integral to membrane	■	■																							
cytoplasm			■			■		■		■	■	■	■		■	■			■	■	■	■		■	■
nucleus				■	■		■		■	■				■				■			■		■		

Figure S1. Gene Ontology (GO) annotations of the genes knocked out in the analyzed studies. Annotations were obtained from the Stanford Source (http://source.stanford.edu) and GeneCards (http://www.genecards.org/), and manually curated. Genes are listed in the same order as the corresponding knockouts in Figure 2 (i.e., in the order of increasing associated variability difference).

S. A. Eraly
