# Supplementary material for: Striking Differences between Knockout and Wild-Type Mice in Global Gene Expression Variability
Source: PLoS One. 2014 May 15;9(5):e97734. doi: 10.1371/journal.pone.0097734 (PMC4022672; doi:10.1371/journal.pone.0097734)
Supplement: Figure S2 — Relative gene expression values of the genes knocked out in the analyzed datasets. Gene expression values of the knocked out genes in (the wild-type replicates of) the tissues in which the corresponding microarray analyses were conducted were obtained as follows: Probe sets on the Affymetrix 430 2.0 microarray corresponding to these genes were identified using NetAffx software (http://www.affymetrix.com/analysis) (with the exception of myostatin which is not represented on this microarray). Most genes were represented by multiple (two to four) probe sets. The relative expression value for each knocked out gene was then computed from data in the corresponding dataset as the average expression in wild-type replicates across all probe sets representing that gene, normalized to the median gene expression value in the dataset. Genes are listed in the same order as the corresponding knockouts in Figure 2 (i.e., in the order of increasing associated variability difference). (PDF) [file pone.0097734.s002.pdf]

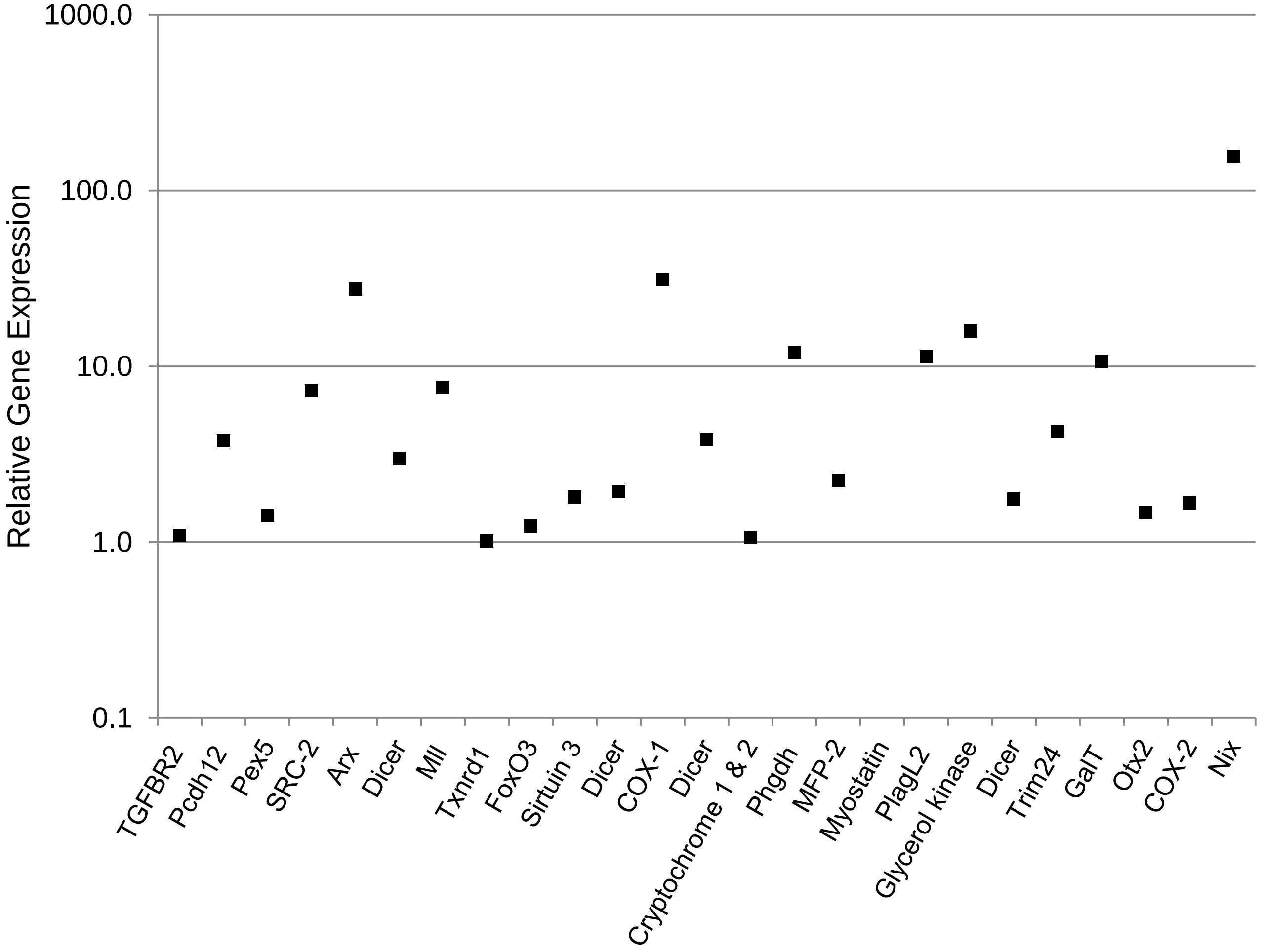

Figure S2. Relative gene expression values of the genes knocked out in the analyzed datasets. Gene expression values of the knocked out genes in (the wild-type replicates of) the tissues in which the corresponding microarray analyses were conducted were obtained as follows: Probe sets on the Affymetrix 430 2.0 microarray corresponding to these genes were identified using NetAffx software (http://www.affymetrix.com/analysis) (with the exception of myostatin which is not represented on this microarray). Most genes were represented by multiple (two to four) probe sets. The relative expression value for each knocked out gene was then computed from data in the corresponding dataset as the average expression in wild-type replicates across all probe sets representing that gene, normalized to the median gene expression value in the dataset. Genes are listed in the same order as the corresponding knockouts in Figure 2 (i.e., in the order of increasing associated variability difference).

S. A. Eraly
