# Supplementary material for: Striking Differences between Knockout and Wild-Type Mice in Global Gene Expression Variability
Source: PLoS One. 2014 May 15;9(5):e97734. doi: 10.1371/journal.pone.0097734 (PMC4022672; doi:10.1371/journal.pone.0097734)
Supplement: Figure S3 — Relationship across the twenty-five analyzed datasets between the mean expression difference between knockouts and wild-types and mean variability difference. The mean expression difference for each dataset was calculated as the mean across probe sets of the difference between log10 mean expression in knockout and log10 mean expression in wild-type (termed here Δlog10mean gene expression (KO-WT)), filtering out probe sets falling below a specified expression threshold. Mean variability differences (termed here Δlog10CV (KO-WT)) were taken from the data in Figure 2 and were calculated in an analogous manner. No significant relationship was noted between the expression and variability differences (Pearson's correlation coefficient = 0.063, p = 0.766; Spearman's correlation coefficient = 0.033, p = 0.87). KO, knockout; WT, wild-type; CV, coefficient of variation. Note the different scales of the two axes. Please refer to the text for details. (PDF) [file pone.0097734.s003.pdf]

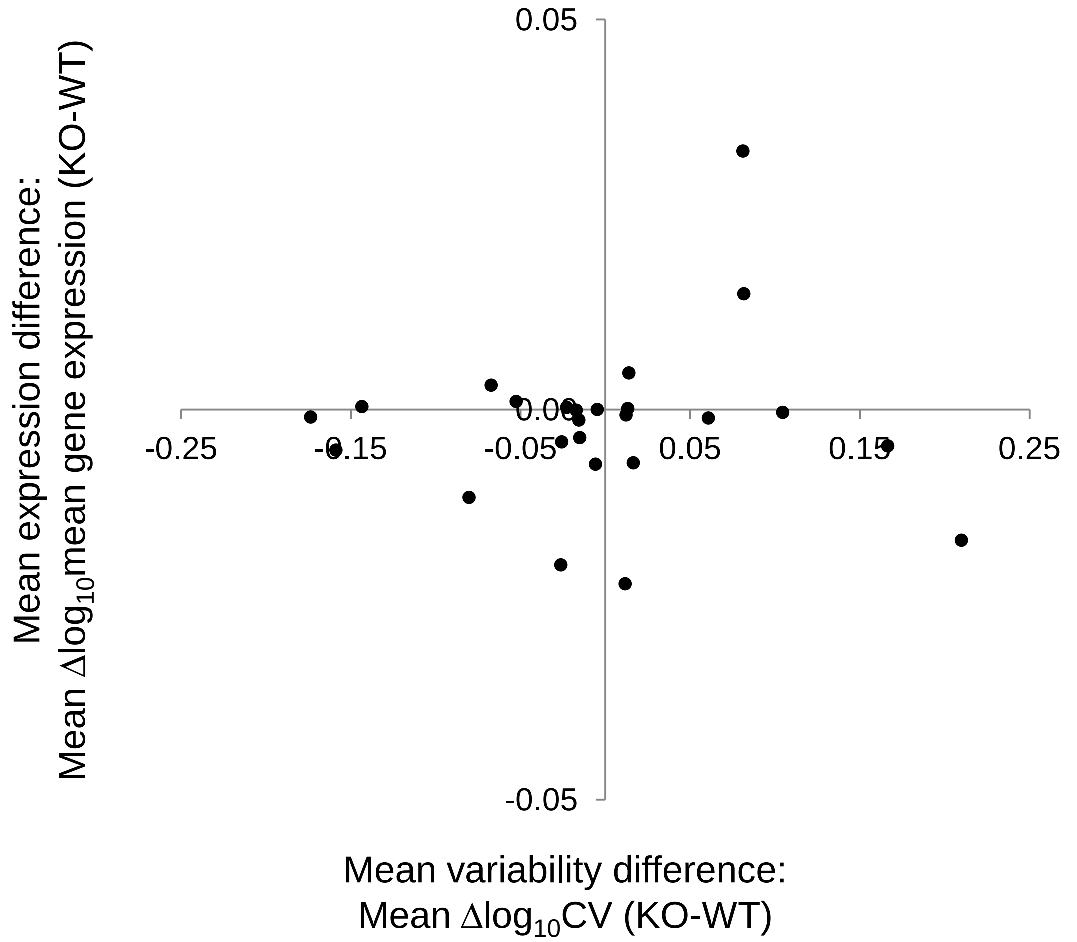

Figure S3. Relationship across the twenty-five analyzed datasets between the mean expression difference between knockouts and wild-types and mean variability difference. The mean expression difference for each dataset was calculated as the mean across probe sets of the difference between $\log_{10}$ mean expression in knockout and $\log_{10}$ mean expression in wild-type (termed here $\Delta\log_{10}$mean gene expression (KO-WT)), filtering out probe sets falling below a specified expression threshold. Mean variability differences (termed here $\Delta\log_{10}$CV (KO-WT)) were taken from the data in Figure 2 and were calculated in an analogous manner. No significant relationship was noted between the expression and variability differences (Pearson's correlation coefficient = 0.063, p = 0.766; Spearman's correlation coefficient = 0.033, p = 0.87). KO, knockout; WT, wild-type; CV, coefficient of variation. Note the different scales of the two axes. Please refer to the text for details.

S. A. Eraly
